# miR-21 Overexpression Promotes Esophageal Squamous Cell Carcinoma Invasion and Migration by Repressing Tropomyosin 1

**DOI:** 10.1155/2020/6478653

**Published:** 2020-10-28

**Authors:** Zhuojian Shen, Xia Xu, Liangzhan Lv, Honglue Dai, Ju Chen, Baishen Chen

**Affiliations:** ^1^Department of Thoracic Surgery, Guangdong Provincial Key Laboratory of Malignant Tumor Epigenetics and Gene Regulation, Sun Yat-Sen Memorial Hospital, Sun Yat-Sen University, Guangzhou 510120, China; ^2^Lung Cancer Research Center of Sun Yat-Sen University, Sun Yat-Sen University, Guangzhou 510060, China; ^3^Department of Cardiothoracic and Vascular Surgery, Tongji Hospital, Tongji Medical College, Huazhong University of Science and Technology, Wuhan 430000, China

## Abstract

The migration and invasion of esophageal squamous cell carcinoma are associated with clinical outcomes, however, the mechanisms remain poorly understood. Here, we found that miR-21 is significantly overexpressed in ESCC, lung cancer, and bladder cancer compared with the adjacent normal tissue. MiR-21 and TPM1 expressions were analyzed by RT-qPCR and WB in 30 ESCC, 10 lung cancer, and 10 bladder cancer clinical specimens, each with matched adjacent normal tissue. Knockdown and overexpression of miR-21 as well as knockdown of TPM1 in ESCC cell lines were performed using synthetic oligonucleotides. TPM1 3′UTR luciferase reporter constructs were used to investigate targeting of TPM1 by miR-21. ESCC migration and invasion were assessed using transwell migration and invasion assays. Inhibition of miR-21 reduced migration and invasion in two ESCC cell lines, and overexpression of miR-21 promoted migration and invasion in vitro. Interestingly, TPM1 exhibited inverse patterns of expression compared with miR-21 in tissues and cell lines. Luciferase reporter assays demonstrated that TPM1 was directly regulated by miR-21. Moreover, the forced overexpression of miR-21 repressed the TPM1 expression, while silencing of miR-21 restored the TPM1 expression in ESCC cell lines. What is more, simultaneous silencing of miR-21 and TPM1 expressions did not alter the migratory and invasive characteristics demonstrating that the effects of miR-21 were mediated through TPM1. In conclusion, the aberrant overexpression of miR-21 is common in cancer and promotes the migration and invasion of ESCC through inhibiting the TPM1 expression. These results suggest that miR-21 may be a novel predictive marker and therapeutic target for treatment of ESCC.

## 1. Introduction

MicroRNAs (miRNAs) are naturally occurring short, noncoding RNA molecules, 21-23 nucleotides long, that regulate the gene expression by binding to mRNAs either to suppress translation or to initiate degradation of target transcripts [1]. Since their initial identification, thousands of miRNAs have been identified in human genome. Some of these miRNAs function as oncogenes, such as miR-31 or miR-21, while others function as tumor-suppressor genes, such as miR-15 or miR-16 [2]. miR-21 has been identified as a central onco-miRNA in tumor formation and is overexpressed in many solid tumors, including esophageal cancer [3–5].

Esophageal squamous cell carcinoma (ESCC) is the most frequent subtype of esophageal cancer and exhibits limited responses to preoperative and postoperative treatment [6]. It is therefore of critical importance to clarify biomarkers as predictive factors or therapeutic targets for the treatment of ESCC. Several biomarkers have been reported to be associated with the development of ESCC, including p53, cyclin D1, and miR-21 [5, 7, 8].

Tropomyosin 1 (TPM1) is a member of the tropomyosins (TPMs), which are actin-associated proteins that play a role in suppressing cancer progression [9]. TPM1 contains a miRNA-21 binding site and acts as a miR-21-mediated tumor suppressor gene in breast cancer [10]. However, little is known about the regulation of TPM1 by miR-21 in the migration and invasion of ESCC. Because miR-21 contains a short seed region (which dictates complementarity to target mRNA transcripts), it is thought that miR-21 has multiple regulatory targets that are associated with cancer progression, such as programmed cell death 4 (PDCD4) [3, 11], TPM1 [10], phosphatase and tensin homologue (PTEN) [12], myristoylated alanine-rich protein kinase c substrate (MARCKS) [13], and maspin [14]. As a regulatory target of miR-21, the role of TPM1 in the progression of ESCC has not been elucidated, particularly when compared with other miR-21-regulated genes. In the present study, we demonstrate that miR-21 regulates ESCC migration and invasion through TPM1.

## 2. Materials and Methods

### 2.1. Cell Lines, Cell Culture, and Human Tissues

ESCC cell lines (EC109/EC1/K30) were obtained from the Key Epigenetic Tumor Laboratory. All ESCC cell lines were grown in RPMI-1640 (Gibco, Gaithersburg, MD, USA), supplemented with 10% fetal bovine serum (FBS) at 37°C in a humidified incubator containing 5% CO_2_. A total of 30 esophageal cancer tissue samples and paired adjacent normal tissue, 10 lung cancer tissue samples and paired adjacent normal tissue, and 10 bladder cancer tissue samples and paired adjacent normal tissue were collected from the Thoracic Surgery Department and Urinary Surgery Department. All tissue samples were stored at -80°C prior to use. The procedures involving clinical tissue samples were approved by the Clinical Medical Ethics Committee.

### 2.2. RNA Extraction and Quantitative RT-PCT Analysis

Total RNA was extracted from cell lines or clinical tissue samples using the TRIzol reagent (Sigma, Santa Clara, California, US). cDNA was synthesized using a Mir-X miRNA qRT-PCR SYBR Kit (TaKaRa, Otsu, Japan) according to the manufacturer's instructions. Quantitative RT-PCR was performed using RealStar SYBR Green qPCR Power Mixture (GenStar, China), and gene expression values were calculated according to the 2^-*ΔΔ*Ct^ method.

### 2.3. Luciferase Reporter Constructs

Two reporters containing the TPM1 plus 3′UTR-WT and the full-length TPM1 plus 3′UTR-mut were purchased from Land Biology (Guangzhou, China). Reporter constructs were amplified from 294 T cells by PCR using the following primers: TPM1XhoIF: 5′CCGctcgagGTTTCTTTGCTTCACTTCTCCC3′, TPM1NotIR: 5′ATAAGAATgcggccgcTAGCTTACACAGTGTTTATTTGACACTG3′, and mutTPM1NotIR: 5′ATAAGAATgcggccgc TTCGAATGACAGTGTTTATTTGACACTGAAAC 3′. The amplified fragments were cloned into a psi-CHECK2 vector (Promega, Adison, WI).

### 2.4. Luciferase Reporter Assay in the ESCC Cell Line

EC109 cells were seeded in 24-well plates at 50-60% confluence and cultivated for 24 hours before transfection. EC109 cells were cotransfected with 1 *μ*L of miR-21 antisense oligonucleotide (20uM) or miR-21 oligonucleotide mimics (20 *μ*M), combined with 0.5 *μ*g of the psi-CHECK2 vectors constructed above, using the lipofectamine reagent following manufacturer's recommendations. Forty-eight hours after cotransfection, the cells were harvested and analyzed using the Dual-Luciferase Reporter Assay System (E1910) (Promega). Cells transfected with a nontargeting oligonucleotide served as a normalization control.

### 2.5. Gene Expression Interference

miR-21 antisense oligonucleotide, miR-21 oligonucleotide mimics, miR-21 scramble, TPM1 antisense oligonucleotide, TPM1 scramble, and TPM1 oligonucleotide mimics ware purchased from GenePharma (Shanghai, China). All transfection procedures were performed using the Lipofectamine 2000 Transfection Reagent (Invitrogen, Carlsbad, California) according to manufacturer's instructions.

### 2.6. Migration and Invasion Assays

Transwell migration and invasion assays were performed in the HST Transwell-24 System (Corning Incorporated, NY). For migration assays, 5 × 10^4^ cells in 0.2 ml FBS-free RPMI-1640 medium were plated in the upper chambers, and 600 *μ*l of RPMI-1640 medium supplemented with 10% FBS was added to the lower chambers. For the invasion assays, 1 × 10^5^ cells were resuspended in 0.2 ml FBS-free RPMI-1640 medium and added into the upper well of Matrigel-coated (BD Biosciences, Canada) invasion chambers. The lower chambers of the invasion assay were filled with 600 *μ*l of RPMI-1640 containing 20% FBS. After 12 hours or 24 hours of incubation at 37°C for migration or invasion assays, respectively, the nonmigrating or noninvading cells that remained on the upper membranes of the inserts were removed by scraping. The cells that had migrated or invaded into the lower chambers and attached to the lower surface of the membrane insert were fixed and stained with a solution of 4% paraformaldehyde and 0.1% crystal violet. Stained cells were imaged and counted using an IX71 inverted microscope (Olympus, Tokyo, Japan).

### 2.7. Western Blotting

RIPA buffer (Sigma-Aldrich, Germany) was used for lysing cells, and total protein was collected by centrifugation at 14,000 × g for 20 min. Protein concentration was determined using a BCA protein assay kit. (Thermo Fisher, USA). Equal amounts of protein lysates were subjected to SDS-PAGE, transferred to methanol-activated PVDF membranes, blocked with 5% nonfat dry milk in Tris-buffered saline (pH 7.4) containing 0.1% Tween (TBST) for 2 h, and incubated at 4°C overnight with primary antibodies (Abcam Biotechnology, UK). Membranes were then incubated with anti-rabbit secondary antibodies (Cell Signaling Technology, China) for 1 h at room temperature. Protein bands were detected using the Pierce ECL Western Blotting Substrate system (Thermo Scientific, Belmont, Massachusetts, US). GAPDH was used as a normalization control for each sample.

### 2.8. Statistical Analysis

Differences between groups were analyzed using Student's *t* test (two-sided), and *P* < 0.05 was considered to indicate statistical significance. Statistical analysis was performed using GraphPad Prism 5.0 (San Diego, CA) or SPSS 13.0 (SPSS Inc., Chicago, IL, USA) software.

## 3. Results

### 3.1. MiR-21 Expression Is Aberrantly High in Multiple Cancers

**Figure 1:**
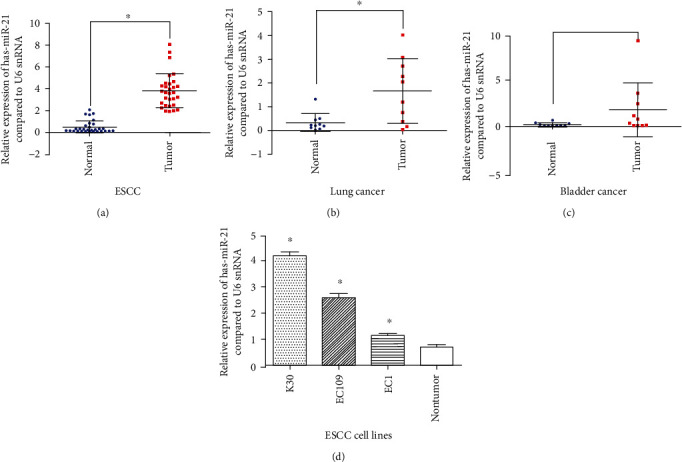
The expression of miR-21 in ESCC, LC, and BC by RT-PCR. (a) The expression of miR-21 in the ESCC cancer tissue and adjacent normal tissue. (b) The expression of miR-21 in the lung cancer tissue and adjacent normal tissue. (c) the expression of miR-21 in the bladder cancer tissue and adjacent normal tissue. (d) The expression of miR-21 in ESCC cell lines and normal tissue (^∗^*P* < 0.05).

To determine the expression status of miR-21in different cancers, a quantitative real-time polymerase chain reaction assay was performed to evaluate the miR-21 expression in 30 ESCC samples, 10 lung cancer samples, 10 bladder cancer samples, and matched adjacent normal tissue. The miR-21 expression was significantly higher in all 30 ESCC samples and in all 10 lung cancer samples compared to paired adjacent normal tissue samples (Figures 1(a) and 1(b)). The 10 bladder cancer samples exhibited higher expression of miR-21 than matched adjacent tissue, but the difference was not statistically significant (*P* > 0.05) (Figure 1(c)). Furthermore, the overexpression of miR-21 was also observed in the ESCC cell lines compared with normal esophagus tissue samples (Figure 1(d)). These results demonstrate that the aberrant overexpression of miR-21 may be a common phenomenon in cancer, especially in ESCC, and suggest that miR-21 may play an essential role in various malignant processes in ESCC.

### 3.2. Ectopic Overxpression of miR-21 Promotes Migration and Invasion of ESCC

**Figure 2:**
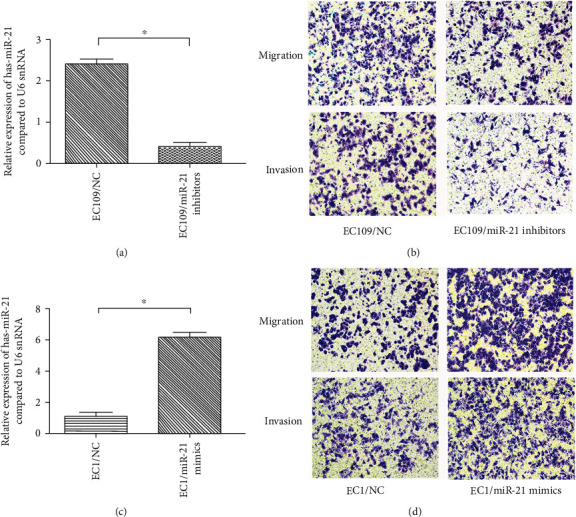
The miR-21 overexpression induces ESCC invasion and migration. (a) miR-21 was detected by RT-PCR after inhibition with miR-21 antisense oligonucleotide in EC109 cells. (b) miR-21 was detected by RT-PCR after the overexpression by miR-21 oligonucleotide mimics in EC1 cells. (c) The transwell assay indicating that inhibition of miR-21 inhibits invasion and migration of EC109 cells. (d) The transwell assay indicating that the overexpression of miR-21 promotes invasion and migration of EC1 cells.

Because miR-21 was commonly overexpressed in ESCC, we performed a gain- and loss-function analysis to address the specific functions of miR-21 on the malignant behavior of ESCC. The EC109 and EC1 cell lines ware transfected with miR-21 antisense oligonucleotide (miR-21 inhibitors) and miR-21 oligonucleotide mimics (miR-21 mimics), respectively, to modulate the intracellular miR-21 expression. Quantitative real-time polymerase chain reaction assay was performed to validate the changes in the gene expression. The miR-21 inhibitors significantly repressed the miR-21 expression in EC109 cells, and the miR-21 mimics resulted in the significant miR-21 overexpression in EC1 cells (Figures 2(a) and 2(b)). Subsequently, transwell assays were performed to test the effects of changes in the miR-21 expression on ESCC migration and invasion. In EC109 cells, antagonism of miRNA-21 significantly impeded migration and invasion (Figure 2(c)). In contrast, transfection with miR-21 mimic promoted migration and invasion of EC1 cells (Figure 2(d)). These data demonstrate that miRNA-21 functions as an oncogene to promote migration and invasion of ESCC cells.

### 3.3. Mir-21 Targets and Downregulates TPM1 in Esophageal Cancer

**Figure 3:**
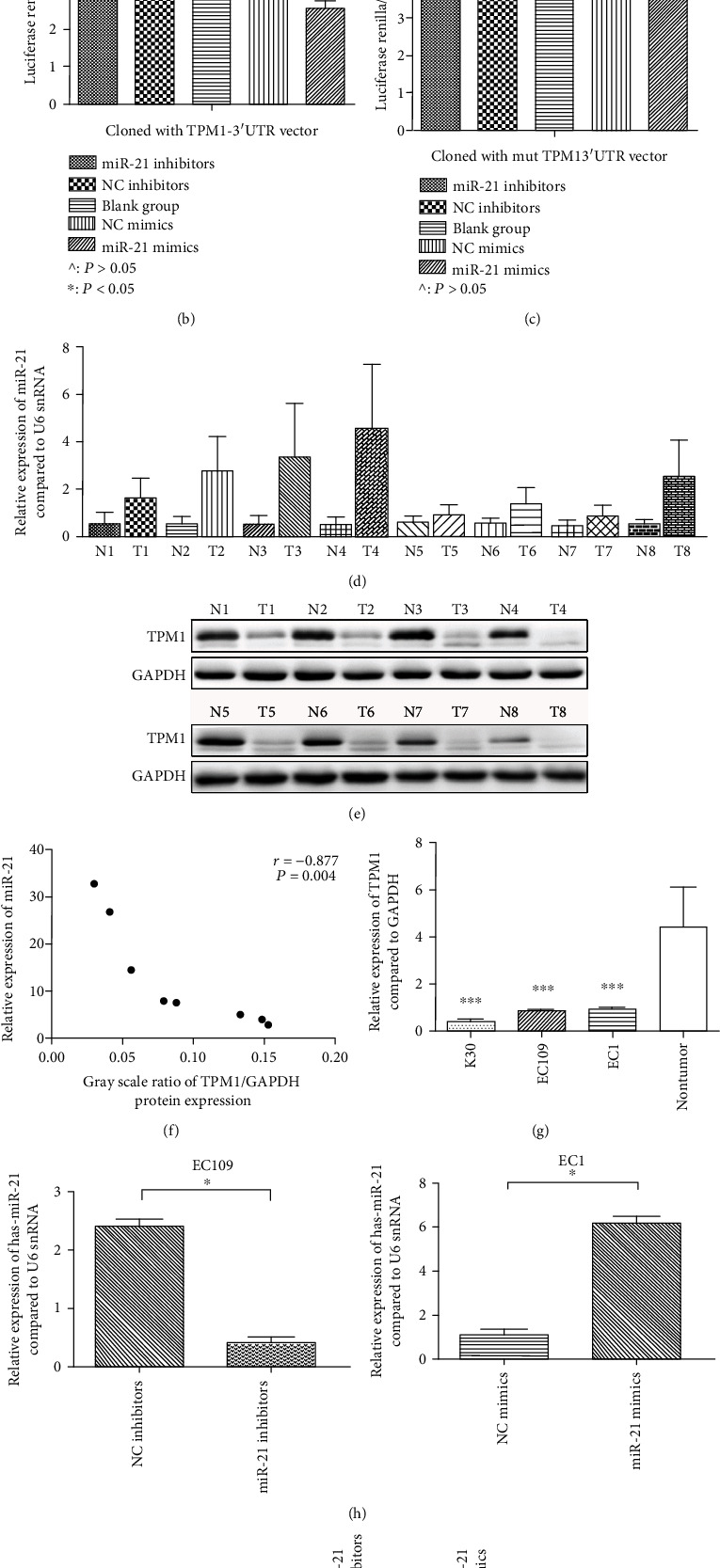
miR-21 targets and represses TPM1 in ESCC. (a) A diagram showing miR-21 binding to its predicted binding site in the TPM1 3′UTR. (b) The relative luciferase activity of the TPM1-3′UTR-WT group after miR-21 interference in EC109 cells. (c) The relative luciferase activity of the TPM1-3′UTR-MT group after miR-21 interference in EC109 cells. (d) The miR-21 relative expression value in 8 ESCC tissues and matched adjacent normal tissues. (e) The TPM1 protein expression in 8 ESCC tissues and matched adjacent normal tissues. (f) The gray scale ratio of the TPM1/GAPDH protein expression and miRNA-21 expression. (g) The expression of TPM1 in ESCC cell lines and normal tissue. (h) The miR-21 relative expression value after miR-21 interference in EC109 and EC1 cells. (i) The TPM1 protein expression after miR-21 interference in EC109 and EC1 cells (*P* > 0.05, ^∗^*P* < 0.05).

Analysis of the TargetScanS miRNA database revealed that TPM1 possesses a putative binding site for miR-21 (Figure 3(a)). This suggests that TPM1, a tumor-suppressor gene related to cancer migration and invasion, is a potential target of miR-21 in ESCC. Luciferase reporter assays were performed in EC109 to determine the suppressive function of miR-21 on TPM1 in ESCC. In EC109 transfected with the TPM1-3′UTR vector (wild type), transfection with miR-21 oligonucleotide mimics significantly reduced the luciferase activity of the 3′UTR-TPM1, compared with the blank group or the negative control double-stranded oligonucleotide group (*P* < 0.05). In contrast, transfection with the miR-21 antisense oligonucleotide increased the luciferase activity from the 3′UTR-TMPI reporter by more than 19% compared with the negative control single-stranded oligonucleotide (*P* < 0.05) (Figure 3(b)). Moreover, in EC109 cells transfected with the TPM1-3′UTR-mut vector, transfection with neither miR-21 oligonucleotide mimics nor miR-21 antisense oligonucleotide had any effect on the luciferase activity compared with the blank group or the negative control double-stranded oligonucleotide group (*P* > 0.05) (Figure 3(c)). These data demonstrate that miR-21 directly regulates the expression of TPM1 in ESCC.

To further confirm that TPM1 is regulated by miR-21 in ESCC, we evaluated the TPM1 protein expression in 8 clinical ESCC samples (including cancer tissues and matched adjacent normal tissues) that had higher miR-21 expression in the cancer tissue compared with the adjacent normal tissue by RT-qPCR (Figure 3(d)). Western blot analysis revealed that the expression of TPM1 was inversely related with the miR-21 expression in these 8 clinical samples, as the TPM1 expression was lower in the cancer tissue compared with the adjacent normal tissue (Figure 3(e)). In these 8 random pairs ESCC samples, the gray scale ratio of the TPM1/GAPDH protein expression was negatively correlated with their miR-21 expression (Figure 3(f)). These data suggest that the repression of TPM1 by miR-21 may be a common phenomenon in ESCC and further suggest that the function of TPM1 may be suppressed by miR-21 in ESCC.

We investigated the expression level of TPM1 in different ESCC cell lines (Figure 3(g)). The expression level of TPM1 mRNA was significantly lower in ESCC cell lines (EC109, EC1, K30) than in adjacent normal epithelial samples (*P* < 0.05). To explore the regulatory effects of miR-21 on TPM1, we transfected the miR-21 antisense oligonucleotide (miR-21 inhibitors) and miR-21 oligonucleotide mimics (miR-21 mimics) into the EC109 and EC1 ESCC cell lines. We verified the miR-21 expression in these cells by RT-qPCR. In accordance with the luciferase assay results, the miR-21 expression was reduced by miR-21 inhibitors in EC109 cells, relative to the negative control inhibitor, and miR-21 was upregulated by miR-21 mimics in EC1 cells compared to the negative control mimics (Figure 3(h)). Moreover, transfection with miR-21 inhibitors significantly increased the TPM1 protein expression in EC109 cells, and transfection with miR-21 mimics significantly reduced or abolished the TPM1 protein expression in EC1 cells (Figure 3(i)). These date further suggest that miR-21 is involved in critical translational regulation of TPM1.

### 3.4. TPM1 Mediates miR-21-Promoted Migration and Invasion in ESCC

**Figure 4:**
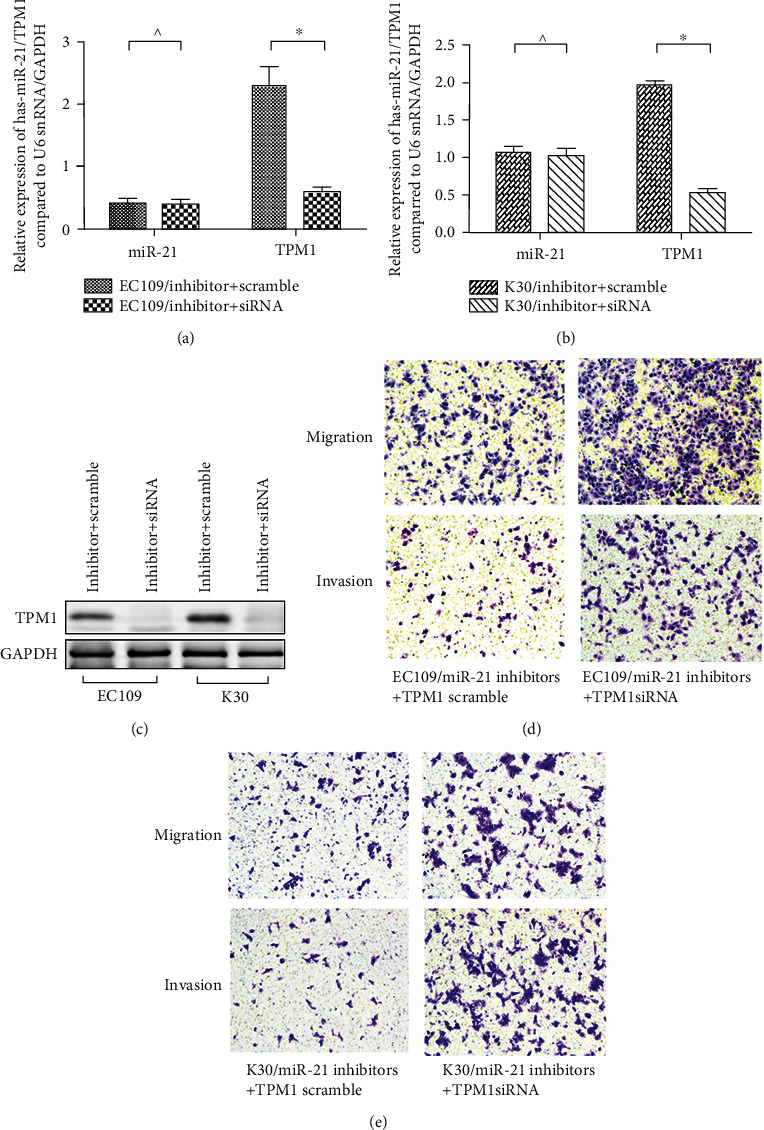
miR-21 promotes ESCC migration and invasion via inhibiting TPM1. (a–c) The effect of cotransfection with miR-21 inhibitors and TPM1 inhibitors was verified by RT-PCR and WB in EC109 and K30 cell lines. (d, e) Invasion and migration of EC109 and K30 cells were evaluated by the transwell assay, showing that invasion and migration were maintained after double silencing of miR-21 and TPM1.

Considering that TPM1 is known as a potent inhibitor of tumor migration and invasion, we determined whether posttranslation silencing of TPM1 is required for miR-21 to promote ESCC migration and invasion. Double transfection of miR-21 antisense oligonucleotide (inhibitors) and TPM1 antisense oligonucleotide (inhibitors) was performed both in EC109 and K30 cell lines, and TPM1 scramble oligonucleotide was used as control. Quantitative RT-PCR confirmed that transfection with TPM1 antisense oligonucleotide effectively repressed the TPM1 expression in both EC109 and K30 cells (Figures 4(a) and 4(b)); significant reduction in the TPM1 protein expression inhibition was also observed (Figure 4(c)). Subsequently, transwell assays were performed in these double gene interference cell line models to determine the effect on migration and invasion. Interestingly, simultaneous depressing of TPM1 and miR-21 rescued the migratory and invasive ability of EC109 and K30 cell lines in which miR-21 was silenced by siRNA (Figures 4(c) and 4(d)). These data suggest that miR-21 induces migration and invasion of ESCC through the repression of TPM1.

## 4. Discussion

In this study, we demonstrate that miR-21 is overexpressed in esophageal squamous cell carcinoma (ESCC), lung cancer, bladder cancer, and in three ESCC cell lines (EC109, EC1, KYSE30); this is an agreement with previous reports [3–5]. Moreover, we report the novel demonstration that miR-21 negatively regulates the expression of TPM1 in ESCC, and that miR-21 induces migration and invasion of ESCC through the translational repression of TPM1.

miR-21 has been widely studied in many cancers [15–19]. Gong and colleagues suggested that miR-21 is an independent predictor of recurrence for phyllodes tumors [17]. In tongue squamous cell carcinoma, the miR-21 expression is an independent prognostic factor of poor survival [18]. The serum miR-21 expression has been shown to be an independent prognostic factor for non-small-cell lung cancer [19]. A relative high ratio of the miR-21 expression in the tumor and normal tissue may be associated with poor survival in ESCC [3]. These reports demonstrate the potential of miR-21 as a prognostic or predictive cancer biomarker.

In this study, we report higher expression of miR-21 in ESCC, lung cancer, and bladder cancer relative to matched normal tissues, highlighting the potential important role of miR-21 in cancer. In the case of ESCC, all 30 ESCC cancer samples expressed high levels of miR-21, while the miR-21 expression was very low or even absent in the matched normal tissue (*P* < 0.05), indicating that miR-21 may be a potent biomarker for ESCC. Indeed, the miR-21 expression in esophageal squamous cell carcinoma is mainly localized in the cytoplasm of stromal cells adjacent to malignant cells [5]. Interestingly, miR-21 may be shuttled between cancer cells and fibroblasts through secreted exosomes [17]. Additionally, miR-21 has also been identified in serum in a remarkably stable form, but exhibits widely variable expression levels [19]. Whether the high expression levels of miR-21 in ESCC tissue may correlate with high serum miR-21 is unknown, further evaluation of the serum miR-21 expression in ESCC patients is warranted. Due to the small sample size and other reasons, the experiment has not yet obtained more clinical significance of miR-21. More clinical sample collections and more comprehensive experiments are needed for the in-depth discussion.

Tropomyosins (TPMs) play an important role in the suppression of metastasis and cancer progression by increasing cell adhesion to extracellular matrix through enhancing actin fibers and focal adhesions [9, 20]. Accordingly, neoplastic transformation has been shown to result in downregulation of the TPM expression [21]. It has been reported that high-grade breast tumor expresses significantly lower levels of TPM1 compared to normal tissues [22]. The role of TPM1 in miR-21-mediated breast cancer cell motility and invasion has been reported [10]. In our study, we found a negative correlation between the TPM1 and miR-21 expression in ESCC. Our data suggest that miR-21 targets TPM1 in ESCC and influences ESCC migration and invasion. We performed dual luciferase reporter assays to confirm that miR-21 suppresses the TPM1 expression of TPM1 by binding to the 3′untranslated region (3′UTR) of the TPM1 mRNA. Finally, in the cotransfection study, we show that miR-21-promoted migration and invasion of ESCC may be mediated through the repression of TPM1.

It is well established that miR-21 can exhibit distinct biological functions dependent on its multiple potential target genes that could be silenced by miR-21 in a given cancer cell type. Silencing of the miR-21 target gene PTEN promotes invasion and migration in ovarian epithelial carcinomas [12], and the repression of the miR-21 target genes TPM1, PDCD4, and maspin can enhance invasion and metastasis in breast cancer [23]. Furthermore, deficiency of the miR-21 target gene PDCD4 is related to proliferation and invasion in ESCC. These various reports demonstrate that miR-21 has a variety of targets in different cancers and suggest that the dominant miR-21-regulated tumor suppressor gene in ESCC has not been confirmed. We used miR-21 inhibitors to upregulate the potential miR-21-mediated tumor suppressor genes in EC109 and K30 cell lines, and we used siRNA to reduce the endogenous TPM1 expression. As a result, when decreasing the endogenous TPM1 mRNA by RNA interference, miR-21 inhibitors could not repress the invasion and migration of these ESCC cell lines. In other words, miR-21 regulates the invasion and migration of ESCC cell lines by targeting the tumor suppressor gene TPM1. TPMs belong to class II tumor suppressor genes that are structurally intact in their gene sequences, but are underexpressed or not expressed due to downregulation or silencing in transcription or translation [24]. With advancements in RNA interference and its clinical application, RNAi-mediated rescue of the silenced TPM1 expression, as well as other potential antioncogenes in ESCC cells with synthetic miR-21 inhibitors, may be a therapeutic method to control ESCC invasion and migration [25–27].

In summary, miR-21 is overexpressed in esophageal squamous cell carcinoma and is negatively correlated with the expression of TPM1. miR-21 regulates the expression of TPM1 by binding to its 3′-untranslated region, and miR-21-promoted ESCC migration and invasion is mediated through the TPM1 regulation. These findings raise the possibility that miR-21 is a potential biomarker to predict ESCC progression, and that miR-21 interference could be an adjuvant therapeutic method for ESCC by inhibiting cancer cell migration and invasion through relieving the TPM1 repression.

## Data Availability

The data used to support the findings of this study are available from the corresponding author upon request.
